# Parametric Material Optimization and Structural Performance of Engineered Timber Thin-Shell Structures: Comparative Analysis of Gridshell, Segmented, and Hybrid Systems

**DOI:** 10.3390/ma19020341

**Published:** 2026-01-15

**Authors:** Michał Golański, Justyna Juchimiuk, Paweł Ogrodnik, Jacek Szulej, Agnieszka Starzyk

**Affiliations:** 1 Institute of Civil Engineering, Warsaw University of Life Sciences, Nowoursynowska 166, 02-776 Warsaw, Poland; michal_golanski@sggw.edu.pl (M.G.); justyna_juchimiuk@sggw.edu.pl (J.J.); agnieszka_starzyk@sggw.edu.pl (A.S.); 2 Faculty of Civil Engineering and Architecture, Lublin University of Technology, 20-618 Lublin, Poland; j.szulej@pollub.pl

**Keywords:** timber structures, parametric modeling, structural optimization, hybrid structures, gridshells, digital fabrication, sustainable design, life cycle assessment

## Abstract

In response to the growing interest in sustainable and material-efficient architectural solutions, this study focuses on innovative applications of engineered timber in lightweight structural systems. It investigates the material optimization and structural performance of engineered timber thin-shell structures through an integrated parametric design approach. The study compares three prefabricated, panelized building systems, gridshell, segmented full-plate shell, and ribbed shell, to evaluate their efficiency in terms of material intensity, stiffness, and geometric behavior. Using Rhinoceros and Grasshopper environments with Karamba3D, Kiwi3D, and Kangaroo plugins, a comprehensive parametric workflow was developed that integrates geometric modeling, structural analysis, and material evaluation. The results show that segmented ribbed shell and two segmented gridshell variants offer up to 70% reduction in material usage compared with full-plate segmented timber shells, with hybrid timber shells achieving the best balance between stiffness and mass, offering functional advantages (roofing without additional load). These findings highlight the potential of parametric and computational design methods to enhance both the environmental efficiency (LCA) and digital fabrication readiness of timber-based architecture. The study contributes to the ongoing development of computational timber architecture, emphasizing the role of design-to-fabrication strategies in sustainable construction and the digital transformation of architectural practice.

## 1. Introduction

In recent years, there has been a dynamic increase in interest in timber structures in response to growing demands for sustainability, climate neutrality, and material efficiency in construction [1,2]. The utilization of technologically processed engineered wood, including but not limited to cross-laminated timber (CLT), laminated veneer lumber (LVL), and plywood, has emerged as a significant substitute for conventional construction materials such as concrete and steel. This development has facilitated the construction of lightweight, prefabricated timber structures that exhibit a reduced carbon footprint [3]. It is evident that analogous conclusions concerning the function of engineered wood in the context of sustainable development and environmental efficiency have been presented in other studies [4]. Contemporary research has confirmed that engineered wood products are characterized by a high strength-to-weight ratio and dimensional stability and the possibility of reuse in circular economy models [5].

Shell structures, encompassing gridshells, segmented shells, and hybrid systems, represent exemplars of advanced timber structures that combine high static efficiency with minimal material consumption [6,7,8]. The employment of double-curved geometry facilitates the transfer of loads primarily through membrane forces, thereby reducing the thickness and weight of the structure by up to 40% in comparison with beam systems [9]. Concurrently, segmented and hybrid timber shells exhibit considerable resistance to local damage, and their assembly and disassembly is uncomplicated, a crucial consideration from a circular economy perspective [10].

It is evident that architectural and structural engineering are currently undergoing a paradigm shift towards a new era of computational timber architecture, a development that has been precipitated by significant technological advances in the fields of parametric design, digital fabrication, and numerical analysis (MES). The integration of form-finding, material optimization, and production data generation in a single coherent environment is enabled by the combination of computational and design tools (e.g., Rhinoceros, Grasshopper, and Karamba3D) [11]. This approach, known as design-to-fabrication, has been shown to reduce execution errors and optimize raw material consumption as early as the modeling stage [12].

The most recent studies indicate a growing need to integrate parametric design with environmental and material analysis. For instance, a cost-effective method has been developed for the fabrication of engineered wood panels with a hexagonal panel layout and half-lap joints, optimized for simple two-axis CNC machines [13]. It has been demonstrated in a number of studies that innovative solutions for joints and configurations of timber elements are of paramount importance, given their capacity to reduce material consumption and facilitate the reuse of components in shell structures [14]. In terms of construction, research on optimizing the placement of fasteners and connections in CLT elements confirms that rationalizing fastening systems can significantly reduce material consumption while maintaining high structural rigidity and durability [15]. Engineered wood structures represent an evolution in the use of timber for improving static and seismic performance [16]. The use of prefabricated wooden panels is not only an economical alternative to reinforced concrete or steel but also a genuinely structural resource [17]. It is appropriate to indicate that dissipative solutions can also be achieved through panels and connections (for example, referring to papers that discuss equivalent damping ratios obtained with suitable steel connections). Robotics and digital material processing techniques are being utilized with increasing frequency in research endeavors concerning prefabrication and automated production of advanced timber structures. Research indicates that the employment of six-axis robots for the shaping and cutting of timber elements facilitates precise joint fitting with minimal material loss, thereby perpetuating the tradition of carpentry in the digital era [18]. Consequently, engineered wood is being elevated to the status of a “digital age” material, whose structural and environmental potential can be fully exploited through the integration of parametric modeling, optimization, and robotic fabrication.

Contrary to the approach of preceding studies, which analyzed individual aspects of the design or behavior of advanced timber structures, this study introduces a novel integrated research approach. This combines geometric, structural, and material analysis in a single parametric model. The proposed methodology facilitates the concurrent optimization of raw material consumption, structural rigidity, and readiness for digital production, constituting a scientific innovation within the domain of research on thin-walled timber shells. As previously evidenced in the extant literature, a paucity of analyses has existed that comprehensively compare gridshells, segmented shells, and hybrid systems in terms of material intensity (kg/m^2^) and elastic deformation. Consequently, the results presented in this paper are the inaugural attempt to quantitatively capture this relationship in a parametric design environment. The developed research model is therefore an original analytical tool that can be used both in engineering practice and in further research on the optimization of sustainable advanced timber structures.

As demonstrated in previous studies conducted by the authors, it is imperative to incorporate numerical analysis, material optimization, and life cycle assessment (LCA) into the design process of engineered timber shell structures [19]. From the perspective of design and structural analysis, the life cycle assessment (LCA) of advanced timber structures is also acquiring increasing importance. Research has demonstrated that the utilization of parametric modeling in Pres-Lam post-tensioned systems facilitates the concurrent optimization of parameters such as stiffness, mass, and CO_2_ emissions throughout the life cycle of a building [11]. These issues have also been developed in recent studies on the integration of parametric modelling and numerical analysis in the design of advanced timber structures [20]. The integration of LCA analysis with parametric design thus opens up new avenues of research into timber architecture, in which form, structure, and environment are considered as a single design system.

To summarize, recent studies suggest that the optimization of thin-walled engineered timber shells can be achieved through an integrated approach that combines parametric design, numerical analysis, environmental optimization, and preparation for digital production. The objective of this study is to develop this issue by comparing three structural systems (i.e., gridshell, segmented shell, and hybrid shell) in terms of material efficiency, stiffness, and prefabrication possibilities. The research was conducted using CAD/CAE tools and a parametric environment (Rhino 8.0 with a latest Grasshopper, Karamba3D 3.1, and Kiwi3D BETA 0.5.0), providing the basis for further methodological analysis presented in the next section.

## 2. Materials and Methods

Dematerialization is a key strategy for sustainable development in the building industry, focusing on using fewer materials and less energy throughout a building’s entire life cycle. This approach aims to minimize negative environmental impacts and reduce the industry’s significant resource consumption. The research project, undertaken by a collaborative team of architects and engineers, creates a systematic, quantitative methodology to optimize material usage for construction of non-standard wooden structures, specifically thin-shells and vaults. This approach aligns with contemporary engineering paradigms that utilize mathematical and statistical methods for data analysis in structural design. Quantitative analysis in this context encompasses descriptive statistics to characterize material properties, correlational assessments to identify relationships between geometric parameters and structural performance, causal-comparative evaluations of different shell systems, and experimental simulations to test hypotheses on material efficiency. The overarching aim is to harness parametric tools for informed decision-making, resource optimization, and elucidation of variable interdependencies, as articulated in the project’s focus on free-form shells for architectural applications.

**Figure 1:**
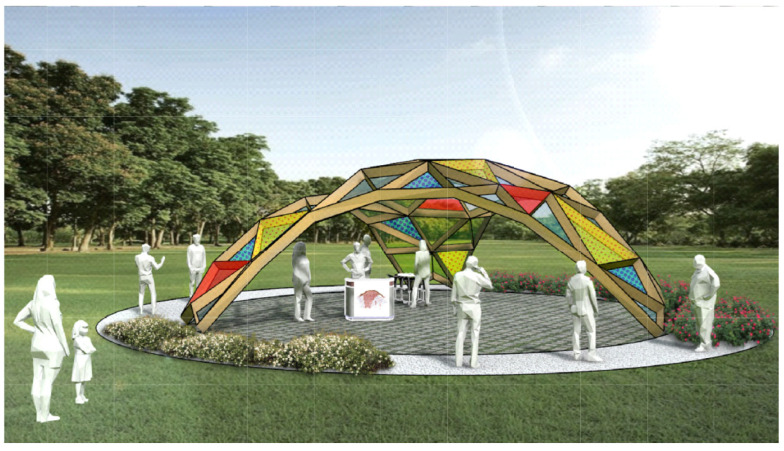
Event pavilion of Warsaw University of life Sciences (SGGW).

The research is largely based on a case study in the form of a concept design of an event pavilion with a structural span of 9.59 m and height of 3.49 m. The Project of Exhibition Pavilion was started by researchers from the University of Zielona Góra as an example of the use of computer numerical form-finding tools in supporting architectural design in the analysis of the effectiveness of structural solutions [21,22]. The project was remodeled and continued as Event Pavilion of Warsaw University of Life Sciences (SGGW) in 2024–2025 with the cooperation of Arch-Eco-lab Sustainable Architecture Research Circle. This endeavor was a part of an ongoing project of Archi-Eco-Lab student research club for SGGW Campus 2030 (Figure 1). The event pavilion is a lightweight prefabricated wooden structure with integrated pro-ecological systems (BIPV, water retention) [23,24,25].

### 2.1. Research Aims and Objectives

The original aim of the research presented in this article is material and resource use optimization aiming to maintain or improve architectural and structural output while using fewer resources and lowering the total energy consumed by materials throughout their life cycle (embodied energy). Development and implementation of a material-oriented methodology for the design of prefabricated engineered wood structures aligns with circular economy principles by promoting restorative systems, eliminating waste by design, and facilitating material reuse and recycling. The second goal is research on design methodologies applying material optimization for such structures.

The primary aim of this article is to advance research in the domain of tools employed for material optimization in lightweight wooden structures, with a focus on parametric approaches to enhance efficiency and sustainability. Central to this exploration is the application of computational Building Information Modeling (BIM) integrated with visual programming (VP) techniques in the research and design of lightweight structures fabricated from structural engineered wood, specifically utilizing 10 mm and 20 mm plywood panels [26,27,28]. This methodology facilitates precise modeling and simulation, enabling iterative refinements in form and material usage. Furthermore, the study validates a novel digital-aided design methodology for wooden structures by leveraging BIM alongside parametric tools, ensuring robustness through empirical testing and comparative evaluations against traditional methods [29,30,31]. The design process methodology is applied across key stages, including form-finding coupled with material and structural optimization to minimize waste and maximize load-bearing capacity; Design for Manufacturing and Assembly (DfMA) principles to streamline fabrication workflows and reduce on-site complexities; and Design for Deconstruction to promote circular economy practices by facilitating easy disassembly and material reuse. Culminating these efforts, the article documents the completion of the conceptual phase for the SGGW pavilion project in Warsaw, demonstrating practical implementation of the proposed systems in a real-world architectural context and providing insights into the comparative performance of gridshell, segmented, and hybrid timber thin-shell structures.

The design methodology adopts a parametric, computational-driven approach rooted in quantitative engineering analysis. This includes experimental simulations using software like Rhinoceros 8.0 with latest Grasshopper plugins (e.g., Kangaroo 2 for physics-based form-finding and Karamba3D 3.1. and Kiwi3D Rhino 8.0 with a latest Grasshopper, Karamba3D 3.1, and Kiwi3D BETA 0.5.0 for finite element analysis) and a DfMA approach in the design process. The method integrates biomimetic principles—drawing from natural forms for efficiency—and topology optimization to minimize material while maximizing stiffness. The literature on engineered wood highlights its anisotropic properties, which are modeled to optimize load paths in shell structures. This aligns with broader trends in sustainable design, where parametric tools enable rapid iteration and performance evaluation.

The final stage of the research, structural analysis, was conducted on a non-standard shell form with resulting form-finding and structural and material optimization. Three variants of the shell structure were compared: discrete gridshell, segmented shell, and ribbed shell combining features of both. All structures were prefabricated using engineered wood-based materials (plywood).

### 2.2. Research on Material Optimization of Engineered Wood Structures

Research results stress that structural and material efficiency cannot be achieved through engineering alone. Veltkamp’s prototypes, such as triangulated spaceframes and tension-net systems, require close coordination between architects, fabricators, and software developers to resolve geometric discrepancies. Charleson documents the rise of “structural artists”—figures like Santiago Calatrava and Cecil Balmond—who dissolve boundaries between disciplines, creating works where load-bearing elements double as sculptural features [32]. Macdonald envisions a future where digital twins and generative AI further streamline collaboration, enabling real-time feedback loops between design and analysis [33]. The selection of materials with minimal environmental impact is a component of environmental certification of buildings (LEED, BREEAM, and WELL) [34,35].

The University of Stuttgart’s Institute for Computational Design and Construction (ICD) and Institute of Building Structures and Structural Design (ITKE) are at the forefront of nature-mimicking strategies, including mechanized creation of timber sheet patterns and auto-adjusting setups like HygroShape. Their joint design tactics for tall wooden edifices stress DfMA via computerized processes and resource-saving approaches such as coreless filament winding, improving ecological soundness, and growth potential [36,37,38,39].

École Polytechnique Fédérale de Lausanne (EPFL)’s Laboratory for Timber Constructions (IBOIS) prioritizes built-in mechanical fixings and parametric strategies for mutual-supporting structures. Their research into joinery techniques and pass-through tenon configurations exploits wood’s inherent qualities for DfMA, aiding swift production of unique pieces and mechanized fitting [40,41,42].

At ETH Zurich, the Arch_Tec_Lab—encompassing Gramazio Kohler Research—progresses algorithm-based and automated manufacturing for intricate timber shapes, as shown in projects like the Sequential Roof, and single-substance barriers. Incorporating DfMA through virtual pre-building and systems like COMPAS FAB allows for flexible connections and recycled material incorporation, demonstrated in novel overhead designs [43,44,45,46].

Further organizations, like the Royal Danish Academy’s Centre for Information Technology and Architecture (CITA), focus on blended arrangements and interchangeable parts for expandable setup [47,48,49]. UCL’s Bartlett School applies parametric simulation for remote DfMA [50,51], whereas Oregon’s TallWood Design Institute examines quake-proof modular building [52]. Aalto University’s Wood Program encourages flexibility with reused wood [53,54,55]. Aarhus’ Emerging Technologies Group creates site-specific automation such as Parawood [56], and Cambridge’s Centre for Natural Material Innovation investigates bendable timber dividers and carbon-neutral high-rise concepts [57,58].

The field of thin-shell design has advanced significantly between 2015 and 2020, particularly in optimizing structural materials like wood, steel, and reinforced concrete to align with circular economy principles such as material efficiency, waste reduction, and resource reuse. In wood-based thin-shell structures, Robeller and von Haaren (2020) introduced the Recycleshell system, utilizing cross-laminated timber (CLT) production waste for polygonal shell elements with form-fitting wood fasteners, enabling wood-only joints that minimize material consumption and support recycling; a 12 m span demonstrator, validated through finite element modeling (FEM) and load tests, demonstrated robust compression performance and architectural versatility for large-span applications with reduced environmental impact [59]. Similarly, Krieg et al. (2015) explored biomimetic lightweight timber plate shells inspired by sea urchins, employing computational design to create segmented structures that achieve high structural efficiency with minimal material use, as evidenced by prototypes that integrate architectural form, strength, and resource optimization [60]. For steel-based designs, Redkin et al. (2016) compared pure steel gridshells to composite steel–concrete variants in high-rise buildings, finding that the composite approach reduces compressive stresses by 20% and deformations by up to 73%, thereby enhancing structural stability and architectural esthetics through improved load distribution and rigidity in a 41-story case study [61]. In reinforced concrete thin-shells, Scholzen et al. (2015) detailed textile-reinforced concrete (TRC) pavilions using shotcrete and carbon fiber, achieving thin-walled efficiency that minimizes material while providing architectural lightness and structural integrity via precise textile placement and erection methods [62]. Kushwaha et al. (2015) conducted a comprehensive SAP2000 analysis on curved beam and grid panel models under various loads, revealing that grid panel slabs offer more accurate and economical results for sustainable thin-shell design, optimizing material use and costs in both architectural and structural contexts [63]. Finally, Hawkins et al. proposed precast TRC shells with prestressed steel ties as alternatives to traditional slabs, demonstrating up to 75% material savings and lower embodied carbon, which supports architectural flexibility and structural performance in multi-story sustainable buildings [64]. These studies collectively underscore the potential of parametric tools in wooden thin-shell optimization, as proposed in the current research, by emphasizing eco-design, modularity, and digital methodologies to reduce embodied energy and promote circularity.

The shift toward free-form architecture in the late 20th century, enabled by computational design tools, challenged traditional structural paradigms rooted in planar geometries [65,66]. Veltkamp traces this transition to the adoption of software from aerospace and automotive industries, which allowed architects to explore complex curvilinear forms but initially lacked corresponding advancements in structural systems [65]. Macdonald contextualizes this progression within broader architectural history, noting that structural innovation has historically been driven by material advancements (e.g., iron, reinforced concrete) and theoretical breakthroughs in understanding form–function relationships [33]. His analysis of structural archetypes—such as arches, trusses, and shells—reveals that efficiency is maximized when geometric configuration aligns with load-transfer mechanisms. Charleson expands this discourse by examining the symbolic and esthetic roles of structure, arguing that efficiency is not merely a technical metric but a design philosophy [32]. Case studies like the Pompidou Center (exposed steel framework) and the Sagrada Família (organic stone vaults) demonstrate how structural elements can simultaneously achieve load-bearing efficiency and cultural resonance. Veltkamp proposes a paradigm shift from static structural typologies to adaptive systems tailored to irregular geometries [66]. By deconstructing free-form buildings into modular components—such as hexagonal or triangular panels—his methodology enables structures to “self-adapt” to local curvature and stress conditions. Parametric modeling tools, including all versions of Rhinoceross-Grasshopper and ANSYS, facilitate real-time adjustments to geometry and material distribution, optimizing structural performance while minimizing waste. Macdonald corroborates this approach, emphasizing that computational tools allow engineers to simulate load paths and identify zones of excess material, thereby refining designs before fabrication [33]. He highlights the importance of finite element analysis (FEA) in quantifying structural efficiency, particularly for shell and spaceframe systems. However, Veltkamp warns against over-reliance on software without manual verification, noting that algorithmic solutions may overlook constructability constraints, such as joint complexity or fabrication tolerances [65,66].

Barbara Misztal, a prominent structural engineer specializing in the theory of structures and construction with a focus on wooden structures, has made significant contributions to the understanding of wooden domes and shell systems, which are foundational for material optimization in thin-shell designs. Her comprehensive work explores historical and modern applications of wooden domes, emphasizing structural integrity, material properties, and innovative construction techniques that align with parametric optimization tools for enhancing material efficiency in thin-shells. In her book *Wooden Domes* co-authored with Misztal and Schmidt, Misztal delves into advanced dome typologies and their material considerations, providing insights into how parametric modeling can refine wooden thin-shell geometries for optimal load distribution [67]. The chapter “Shell Domes” examines the mechanics of curved wooden surfaces, highlighting material behaviors under stress that inform parametric algorithms for minimizing material usage in thin-shell structures [68]. Similarly, “Ribbed Domes” discusses ribbed configurations in wooden constructions, offering data on stiffness and elasticity that can be parameterized to optimize material selection and reduce waste [69]. Misztal’s analysis in “Selected Examples of Domes from Glued Laminated Timber” showcases practical implementations of laminated wood in domes, demonstrating how parametric tools can simulate and improve material performance in thin-shell applications [70]. The section on “Gridshell, Ribbed–Shell Domes” addresses hybrid shell forms, emphasizing grid-based optimizations that align with computational design for material-efficient wooden structures [71]. In “Multi-Shell Domes,” she explores layered shell systems, providing historical and technical bases for parametric variations in material thickness and composition to achieve lighter yet robust thin-shells [72]. Her recent paper *On the Variability in Time of the Longitudinal Modulus of Elasticity E and the Traverse Modulus of Elasticity G and Their Impact on the Rigidity of Timber Structures* investigates time-dependent material properties of wood, crucial for parametric modeling in predicting long-term performance of optimized thin-shell structures [73]. Finally, “Selected Elements of the Dome Building History” contextualizes evolutionary material choices in domes, underscoring the role of parametric tools in modernizing traditional wooden thin-shell engineering [74].

### 2.3. Research Gap

The article addresses a key research gap in material and structural efficiency by demonstrating how parametric tools can enhance energy efficiency during the prefabrication chain for engineered wood materials like plywood, an area underexplored in recent studies focused primarily on general timber optimization without specific CNC integration. It fills the gap in prefabrication processes by providing empirical evidence on reducing embodied energy through optimized CNC processing of thin wooden shells, building on limited 2020–2025 publications that emphasize cost-effectiveness but overlook detailed material flow efficiencies. In eco-design and optimization, the article bridges the shortfall in applying geodesic-inspired strategies to wooden thin shells, showing how parametric design achieves maximal structural strength with minimal material use, extending beyond biomimetic approaches in the existing literature. The research closes the gap in sustainable design by integrating circular economy principles into thin-shell optimization, where prior works from 2024 to 2025 on segmented timber shells discuss relocation but lack comprehensive material reuse metrics for lightweight structures. For modularity and adaptability, the article fills a void by proposing separable gridshell and infill components in prefabricated wooden shells, facilitating refurbishment and longevity, which recent modular construction reviews highlight as underutilized in timber applications. It addresses the adaptability gap by emphasizing easy disassembly for reuse in engineered wood structures, contrasting current studies on disassembly in building design that do not specifically tackle thin-shell modularity challenges.

In digital design, the article remedies the limited integration of BIM for precise tracking in wooden prefabrication, as evidenced by its methodology for thermal and strength modeling, an advancement over scattered 2020–2025 publications on digital tools without focused wood shell applications. The research fills the gap in human–machine interaction by incorporating Augmented Reality (AR) in the prefabrication process for thin wooden shells, enhancing decision-making where recent circular economy scoping studies (2024) mention digital engineering but omit AR specifics. It bridges the data-driven resource efficiency gap by combining BIM and parametric tools for real-time monitoring in engineered wood processing, addressing shortcomings in 2025 conference papers on circular building that prioritize broader scales over prefabricated thin shells. Finally, the article comprehensively fills cross-area gaps by linking material efficiency, eco-design, modularity, and digital tools in a unified methodology for wooden thin shells, promoting circularity in ways that the fragmented recent literature (2020–2025) on timber structures has yet to fully achieve.

### 2.4. Detailed Research Process

**Figure 2:**
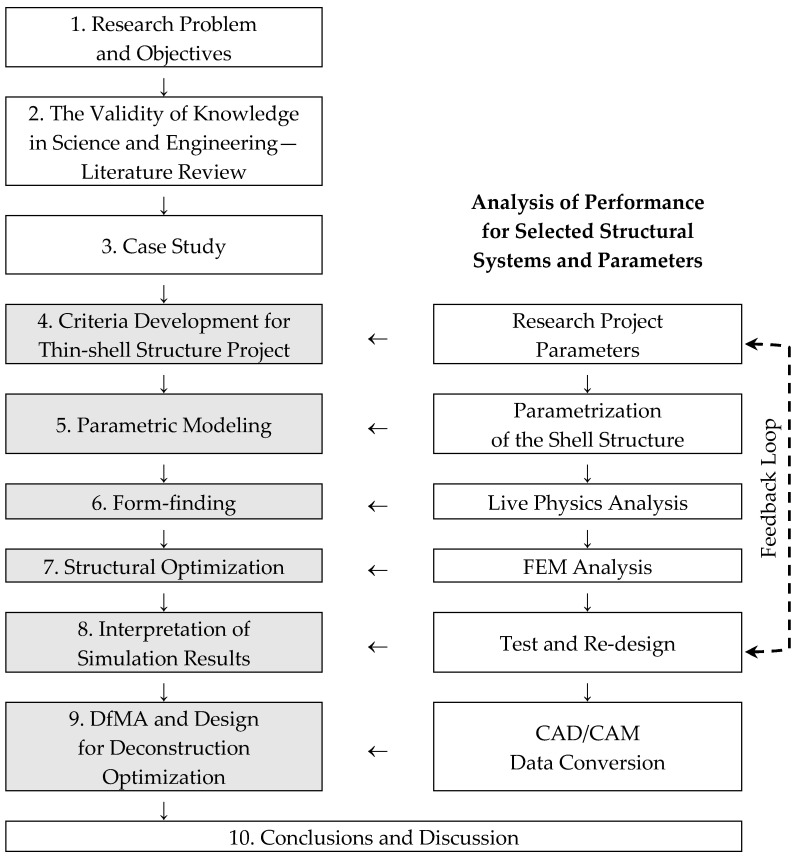
Methodology flowchart for research process for material and structural optimization of engineered wood thin-shell structures.

The research process is structured into five iterative stages, executed within a computational environment using Rhinoceros (Rhino) software augmented by Grasshopper for visual scripting. This parametric framework allows dynamic adjustment of variables, facilitating optimization loops. Plugins such as Kangaroo Physics enable real-time simulations of physical behaviors (e.g., tension and compression), Karamba 3D performs finite element analysis (FEA) for stress distribution, and Kiwi3D supports advanced isogeometric analysis for shell behaviors. The stages are as follows (Figure 2):

Experimental research was carried out using the Rhinoceros program with the Grasshopper application. This is a popular modeler today, based on Non-Uniform Rational B-spline (NURBS surfaces). These are parametric equations graphically based on topology. This is important, especially when dealing with, for example, dividing surfaces in a consistent manner, independent of its global geometry or the given pattern, while preserving the designer’s intent. Experimental research involved virtual prototypes subjected to simulated loads, aiming for optimal shell forms minimizing structural material consumption. Criteria included material intensity (e.g., 35 kg/m^2^ target) and stiffness (deflection limits L/300). Using Rhino/Grasshopper ecosystems, iterations yielded variants, with hybrids excelling due to integrated load-sharing. This mirrors precedents like the livMatS biomimetic shell, where segmented designs achieved 41% weight savings. Quantitative outcomes were statistically analyzed for correlations, revealing hybrid solutions’ potential for 30–60% efficiency gains.

The results of geometric analyses were used to conduct a research experiment in the field of parametric modeling to select the most advantageous variant in terms of material consumption. In a broader perspective, undertaking, implementing, and evaluating the research allows for the development of modeling methods that enable achievement of the best results in terms of attaining the buildability of parametric models at the architectural concept stage.

### 2.5. Efficient Structural Systems

Shell structures, characterized by their thin, curved surfaces, derive strength from double curvature, which mobilizes membrane stresses to resist loads. Angus J. Macdonald traces this concept to natural analogs like eggshells and sea urchins, where curvature enhances rigidity despite minimal material thickness. Structural systems, such as form-active and surface-active structures, maintain a strong relationship between form and structural behavior, such as that exemplified in works of most innovative 20th century structural engineers: Félix Candela, Eduardo de Toroja y Miret, Buckminster Fuller, Oscar Niemeyer, Frei Otto, Werner Sobek, Heinz Isler, and Luigi Nervi. The hyperbolic paraboloid, employed in Félix Candela’s concrete shells, demonstrates how anticlastic curvature (opposite directional curves) mitigates buckling while enabling dramatic cantilevers. Design strategies for those structural systems focus specifically on compression shells and tensile structures such as membrane structures [75].

The TWA Terminal at JFK Airport, analyzed in *Structure As Architecture*, showcases how Eero Saarinen’s wing-like concrete shells embody aerodynamic elegance while functioning as load-bearing roofs. Charleson notes that such designs challenge the dichotomy between structure and ornament, as the shell’s form is inextricable from its structural logic [32].

Early free-form structures often relied on conventional frameworks, such as rectilinear steel frames, which were ill-suited to irregular geometries and led to inefficiencies in material usage [76]. Advances in computational design have expanded the possibilities of shell structures beyond conventional geometries. Non-standard shells, often featuring minimal surfaces or algorithmic patterning, optimize material usage while accommodating programmatic complexity. The Zaha Hadid-designed Heydar Aliyev Center in Baku employs a continuous steel, spaceframe-clad, in-glass-reinforced concrete, its sinuous form achieving structural stability through varied curvature radius [77].

A prerequisite for the design and construction of structure types selected for the research project is a solid knowledge of their structural behavior [78]. Initial modeling incorporates biomimetics (e.g., emulating bone lattices for efficiency, crystallography) to redistribute material for minimal weight under constraints [79]. Macdonald highlights that such designs rely on finite element analysis (FEA) to simulate stress distributions and deformations, a process exemplified in Martínez Valle et al.’s curvilinear coordinate FEM method for thickness-independent shells. By integrating shear deformation theories and mixed interpolation techniques, modern shell analysis circumvents locking effects, enabling accurate modeling of both thin and thick configurations [33].

A gridshell is a form- and cross-section-active, lightweight structure, composed of discrete members connecting nodal points following a curved shape. Studies on gridshells emphasize elastic deformation of timber laths for double-curved forms, with material optimization achieved through dynamic relaxation algorithms. Thin-shell designs leverage wood’s compressive strength, often using cross-laminated timber (CLT) for spans up to 48 m, as seen in prototypes reducing weight by 41% via parametric panelization. Segmented shells promote circularity through reusable cassettes, while hybrids combine elements for enhanced efficiency, potentially lowering the environmental impact by 30%. These findings underscore the research’s contribution to parametric development in architectural–structural collaboration. Gridshells epitomize the fusion of free-form esthetics with structural rationality. Comprising interconnected linear elements arranged in a lattice, gridshells gain rigidity through double curvature, akin to traditional shells but with greater formal flexibility [80,81,82]. M. Veltkamp’s *Free Form Structural Design* elucidates how parametric modeling enables gridshells to self-adapt to local geometric and load conditions, ensuring optimal force distribution without compromising fabrication simplicity [65]. The Savill Building in the UK, with its undulating timber gridshell, illustrates this principle: elastic deformation during construction allowed flat laths to assume a complex curvature, locked in place through tensioning [80]. Unlike geodesic domes, which are constrained to spherical tessellation, gridshells accommodate asymmetric and non-uniform curvatures. This adaptability, as Veltkamp argues, aligns with contemporary architectural trends toward fluid, non-repetitive forms [65,66].

Spaceframes, three-dimensional truss systems, combine the lightness of skeletal structures with the spanning capacity of shells. Composed of tetrahedral or octahedral modules, spaceframes distribute loads multidirectionally, making them ideal for large-span roofs like stadiums and airports. Charleson cites Norman Foster’s British Museum Great Court Roof as a paradigm, where a steel spaceframe spans 7100 square meters with minimal intermediate supports, its triangulated nodes resolving complex force interactions. Veltkamp notes that spaceframes’ modularity allows prefabrication and rapid assembly, reducing construction timelines. However, their geometric regularity contrasts with free-form shells, necessitating compromises between structural efficiency and formal innovation [32].

Material-efficient wooden structures, such as cross-laminated timber (CLT) and mass timber, are increasingly adopted by major architects for their sustainability and structural advantages in modern designs. Architects like Michael Green have pioneered the use of these materials in large-scale projects, emphasizing reduced material waste and enhanced efficiency compared to traditional building methods [83,84]. Examples include innovative buildings that incorporate engineered wood for faster construction and lower environmental impact, as seen in award-winning structures recognized by organizations like WoodWorks [85]. These approaches allow for lightweight yet durable frameworks, enabling architects to create multifunctional spaces with minimal resource consumption. Overall, the trend reflects a shift toward eco-friendly architecture, with firms redefining wooden building techniques to meet contemporary demands for efficiency and esthetics [83,84].

### 2.6. Research Methodology

The adopted research methodology draws from interdisciplinary precedents where integrative parametric workflows optimized segmented timber cassettes for a 30% reduction in environmental impact (Table 1). Similarly, asymptotic geodesic hybrid gridshells utilize planar plywood elements for fabrication simplicity, reducing waste in wooden structures. At SGGW, the focus on wood technology—encompassing hydrothermal treatment and structural physics—underpins the experimental design, extending prior work on compression strength in wood composites. The research presented in this article aims to determine a design methodology for prefabricated, lightweight structures constructed with structural engineered wood (plywood, LVL). Integration of architectural and engineering design is crucial in achieving structural and material efficiency. However, material-based design methodology with CAD, BIM, and programming is organized around the core, which is a parametric model used for load simulations in early structural design. The case study structure is modeled on recent examples of experimental, lightweight pavilions made of engineered wood. They are presented in Table 1.

**Figure 3:**
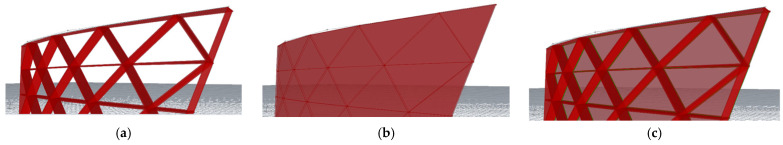
Structural systems examined in research: (**a**) discrete gridshell; (**b**) segmented shell made of engineered flat wood panels; (**c**) hybrid ribbed shell system.

The research presented in this article comprises an early structural design study in which the three following engineered timber thin-shell structural systems—discrete gridshell, segmented shell, and ribbed shell (hybrid)—were comparatively analyzed for their structural and material efficiency through parametric optimization and performance simulations under uniform loading conditions (1.9 kPa). The three evaluated systems are illustrated in Table 2 and Figure 3.

The discrete gridshell, characterized by elastic deformation of timber laths into double-curved lattices with orthogonal or diagonal meshes, exhibited moderate stiffness with deflections ranging from 2 to 5 mm per meter of span, relying primarily on curvature for stability, and a material efficiency of 40–60 kg/m^2^, as exemplified by the Mannheim Pavilion (1975) with its 50 mm × 50 mm hemlock laths spanning 60 m [96,97]. In contrast, the segmented shell, utilizing modular planar panels such as cross-laminated timber (CLT) cassettes assembled with reversible joints, demonstrated higher stiffness with deflections under 2 mm per meter, enhanced by topology optimization, and an improved material efficiency of 25–35 kg/m^2^, as seen in the BUGA Wood Pavilion, which achieved a 41% weight reduction compared to solid CLT through hollow laminated veneer lumber (LVL) cassettes. The ribbed shell (hybrid) system, integrating gridshell lattices with segmented panels or bracing via reciprocal nodes, outperformed both in efficiency, offering superior stiffness (2.3–2.4 mm deflection under a 300 kg loading) and the lowest material use at 20–30 kg/m^2^, representing up to a 60% reduction, as illustrated by the Reciproframe Gridshell with its plywood frames, spruce bracing, and robotic fabrication [92]. These findings highlight the hybrid system’s potential for optimizing resource use in large-span timber structures while maintaining robust performance [98].

### 2.7. Material Optimization and Structural Performance of Plywood

Plywood made from birch is one of the most popular options within the 670–750 kg/m^3^ density range, valued for its strength, smooth finish, and versatility in applications like furniture and cabinetry. Oak plywood also falls commonly in this density bracket, offering durability and an attractive grain pattern suitable for flooring and structural uses. Regarding orthotropic properties, plywood exhibits unique mechanical characteristics in three perpendicular directions due to its layered veneer construction, with strength and stiffness varying along the grain (longitudinal), across the grain (radial), and through the thickness (tangential), making it more predictable than solid wood in engineering designs. [99,100]

When comparing the density of 10 mm and 20 mm plywood, there is no significant difference, as both typically range from 670 to 750 kg/m^3^, maintaining consistency across various thicknesses (Table 3). However, the weight per square meter effectively doubles with the increase in thickness, resulting in approximately 6.7–9 kg/m^2^ for the thinner variant versus 13.4–15 kg/m^2^ for the thicker one, which can influence handling and transportation in structural applications [21,101,102].

For the modulus of elasticity (MOE) in bending, the 20 mm plywood exhibits values that are 10–15% lower than those of the 10 mm version, with ranges of 8700–11,000 N/mm^2^ compared to 10,000–12,000 N/mm^2^, primarily due to the increased number of layers in thicker sheets. Despite this, the overall stiffness (EI) for 20 mm plywood is substantially higher—approximately 3–6 times greater—owing to enhanced section properties, making it more suitable for deflection-resistant uses like flooring or roofing [21,101,102].

Bending strength shows a similar trend, with the 20 mm plywood having 8–13% lower specific strength (48–49 N/mm^2^) than the 10 mm (53–57 N/mm^2^), yet its absolute bending capacity is 2–3 times higher due to the greater thickness, enabling better performance under load in beams or panels [103,104].

Shear strength in panel shear remains constant at around 10.0 N/mm^2^ for both thicknesses, with negligible variations in rolling or planar shear (about 2.4 N/mm^2^ for 10 mm versus 2.3 N/mm^2^ for 20 mm), indicating that intrinsic material shear properties do not scale with thickness [21,101,102].

In terms of shear capacity, the 20 mm plywood offers 2–3 times the capability of the 10 mm (27–33 kN/m versus 15–21 kN/m), as it scales linearly with the cross-sectional area, providing superior resistance in shear-critical scenarios such as wall bracing or diaphragms [21,101,102].

Tension strength is 4–6% lower for the 20 mm plywood (41–42 N/mm^2^) compared to the 10 mm (43–45 N/mm^2^), but the overall tension capacity per meter width is about 1.7 times higher for the thicker option, benefiting tensile-loaded elements like trusses [21,101,102].

Compression strength base values are consistent across both thicknesses, typically ranging from 20 to 45 MPa depending on the grade (e.g., F8 to F17), with the 20 mm providing roughly 1.7 times the capacity per meter due to increased area, which is advantageous for compressive structural members [21,101,102].

Finally, bending capacity for an F8 grade example is 3–4 times higher in the 20 mm plywood (950–1288 N/mm width) than in the 10 mm (250–363 N/mm width), driven by the larger section modulus, underscoring the thicker plywood’s preference for high-moment applications [21,101,102].

## 3. Results

The process, conducted by architects and engineers at Warsaw University of Life Sciences (SGGW), emphasizes quantitative experimental approaches, including descriptive and correlational analyses, to evaluate structural performance and inform design strategies for architectural applications of engineered wood projects.

The results underscore hybrid shells’ advantages in wooden structures, offering stiffness comparable to CLT with reduced mass, as validated by FEA and prototypes. Discussions formulate conclusions on non-standard modeling, advocating parametric integration for sustainable design. In broader terms, this advances parametric modeling in architecture, emphasizing collaboration and digital fabrication. Future directions include real-scale testing and anisotropic modeling enhancements.

**Figure 4:**
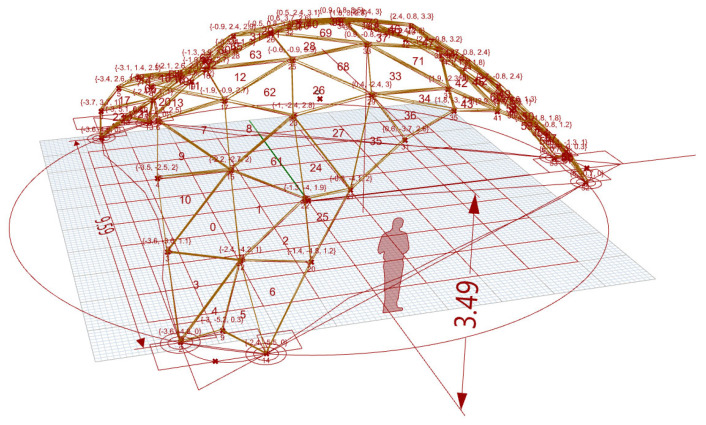
Form-finding. Numerical model of the covering defined by means of a polygon mesh spread over a minimal surface.

**Figure 5:**
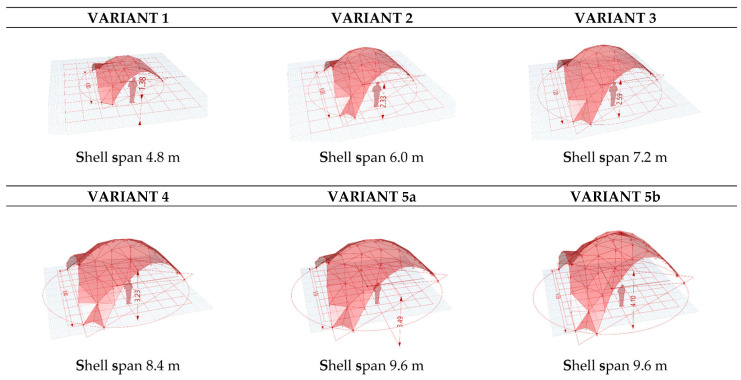
Form-finding process. Parametric model iterations generated by the Kangaroo BouncySolver component.

Structural optimization of form is achieved through Live Physics and FEM simulations of every structural type represented in parametric NURBS and discrete mesh models (Figure 4). Parametrization of the structural form model, including minimal surfaces (zero mean curvature geometries) and catenary curves (gravity-optimized arches), informs the parametric model, generated in Rhino/Grasshopper with Kangaroo for the form-finding module [105]. This stage establishes a flexible parametric mesh model, allowing variations in curvature and load paths, akin to methods in hygromorphic wood gridshells where self-shaping bilayers achieve double curvature (Figure 5).

Form-finding through Live Physics analysis of the parametric model aids optimization [22]. Structural and material efficiency strategies target building statics, employing line-of-thrust analysis to ensure compressive forces remain within the geometry and avoiding bending moments. The first step is free-form surface tessellation, which divides curves into meshes using Weaverbird, with Kangaroo simulating equilibrium. This quantitative step uses descriptive statistics to evaluate efficiency metrics, such as effective bending stiffness (EI_eff) and shear modulus (GA_eff), as per standards like CSA O86 (Figure 5).

Three structural systems have been comparatively assessed: segmented gridshells with modular and interconnected panels (with no panel infills), segmented shells (with planar modular panels), and hybrid shells (combined shell and ribs). Orthotropy of the panel was considered in FEM analysis and IGA, differentiating behavior in one direction versus its perpendicular. The results of the FEM analysis are listed according to structural and material efficiency. Simulations in Karamba and Kiwi3D evaluate stiffness and material consumption under loads (e.g., self-weight, snow) [106,107].

**Figure 6:**
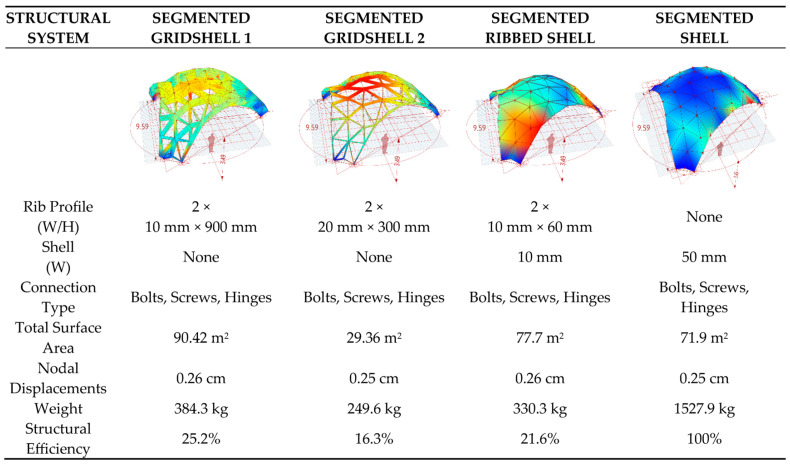
Structural parameters and FEM analysis results (Karamba 3D, Kiwi 3D).

The study revealed a higher efficiency in employing plywood profiles with enhanced sheet stiffness (20 mm thickness) compared to the variant utilizing flexible sheets (10 mm). Furthermore, the continuous, full-plate plywood shell variant demonstrated low efficiency coupled with high material consumption. In contrast, the hybrid solution, comprising a shell reinforced with a ribbed gridshell, exhibited high efficiency (Figure 6).

Final CAD/CAM data conversion translates parametric data into manufacturing instructions using native Grasshopper and tools like WoodBee (timber processing), Sawfish (cutting paths), and RoboDK (robotic simulation). This ensures precision, with experimental validation confirming sub-millimeter accuracy. Design for Manufacturing and Assembly (DfMA) for digitally assisted CNC manufacturing refines the parametric model in Rhino/Grasshopper, ensuring compatibility with multiple-axis laser cutting or robotic milling. This stage incorporates causal-comparative methods to compare fabrication variants, prioritizing reversible joints for circularity.

Research suggests that parametric modeling, incorporating biomimetics and topology optimization, enables significant material savings in wooden shell structures, potentially reducing consumption by up to 60% compared to traditional methods, though outcomes vary based on system type and fabrication precision. With tools like Rhinoceross/Grasshopper facilitating iterative analysis, evidence leans toward hybrid ribbed shell offering the highest efficiency in stiffness and material use and functional roofing, with the gridshell structure being the most efficient timber structure.

The evolution of architectural design toward free-form geometries has necessitated parallel innovations in structural engineering to ensure both esthetic integrity and technical performance. This review synthesizes insights and seminal texts to evaluate how contemporary research addresses structural efficiency (optimizing load-bearing capacity relative to material use) and material efficiency (minimizing waste and embodied energy). These works collectively emphasize the interdependence of form, function, and fabrication, advocating for systems that harmonize architectural ambition with engineering rigor.

A comparison of the results obtained in this study with data from the literature reveals that the reduction in material weight in the hybrid shell system (30–50% compared to a segmented solid shell) is consistent with the range of reductions reported in the study by Hua et al. (2020) [108]. In that study, the use of the Voronoi structure reduced timber consumption by 45% while maintaining the load-bearing capacity of the structure [108]. These results indicate that the control of the topology and spacing of elements in a parametric model is a pivotal factor influencing material efficiency.

In the analysis of maximum displacements, it was noted that gridshell shells are more susceptible to deformation, a finding that is consistent with the results of more recent experimental and numerical studies by Schling, Wan, and D’Acunto (2023) [109]. These researchers proved that double curvature and control of the bending radii of elements significantly increase the stiffness and stability of timber shells [109].

The numerical outcomes thus substantiate the hypothesis that optimizing curvature in a parametric model can serve as an efficacious instrument for augmenting stiffness with constrained raw material utilization. Concurrently, it was observed that the geometric efficiency of the structure is enhanced when internal forces are predominantly transferred by membrane compression, in accordance with the principle of form follows force [110].

**Figure 7:**
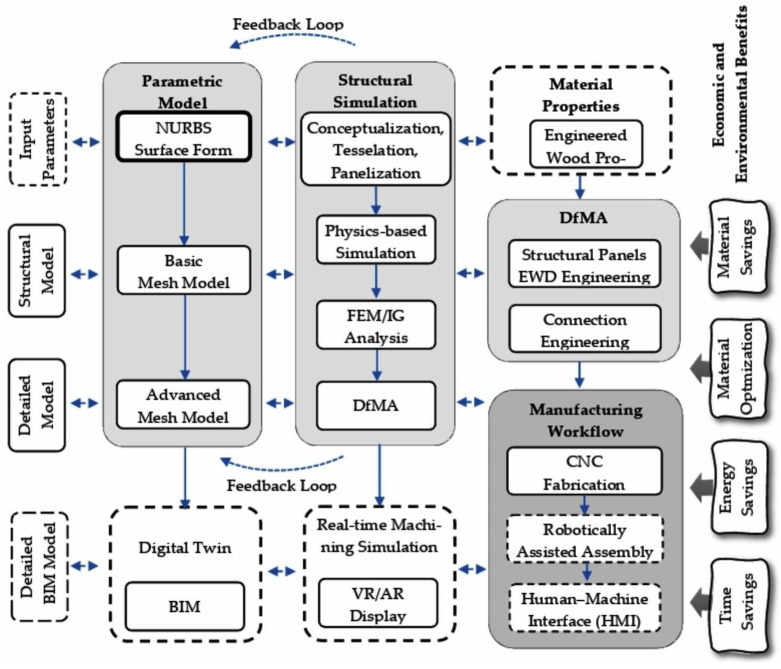
Structural and material efficiency through integrated architectural and engineering design.

Integration of architectural and engineering design is important in developing tools for structural and material efficiency. CAD, BIM, and parametric programming are the tools in the creation of a parametric model, which becomes core when all the stages (conceptual and target) are linked in a feedback loop (Figure 7).

The parametric tools employed in the research facilitated comprehensive integration of the form-finding stage, structural analysis, and preparation for digital production. The findings of this study are in alignment with the observations made by Adelzadeh et al. (2023) [93], who developed the ReciprocalShell system. This system is a hybrid prefabricated timber shell model that is based on modular geometry and reversible nodes, resulting in a reduction in material waste of more than 25% [93]. In accordance with the research conducted by Porras et al. (2024) [13], this study corroborates the finding that the integration of CAD and MES tools enables the direct generation of data for CNC machines. This process has been shown to result in a substantial reduction in the duration of the design and production process, as well as a significant decrease in material wastage [13].

The model developed in this study can therefore be regarded as a tool consistent with the Design for Manufacturing and Assembly (DfMA) concept, enabling the design of structures that are optimized not only structurally but also in terms of production. From an engineering practice perspective, this is important for the development of a new generation of lightweight, sustainable architectural structures.

## 4. Discussion

The analysis, which was conducted for three categories of thin-walled shells, namely, gridshell, segmented shell, and hybrid ribbed shell, demonstrated clear disparities in material efficiency and structural stiffness. The employment of an integrated parametric approach (Grasshopper, Karamba3D, Kiwi3D) facilitated a quantitative assessment of these relationships, founded upon a uniform geometric and numerical model. The findings demonstrate that optimizing geometry and structure enables a substantial reduction in material usage while preserving high stiffness, thereby validating the efficacy of the parametric design methodology employed (Figure 7). The subsequent discussion in this paper explores the implications of these findings within the broader context of existing research, particularly in relation to the design and fabrication of sustainably engineered materials.

A comparative analysis demonstrated that hybrid shells attained the optimal material efficiency ratio, exhibiting a combination of minimal weight with advantageous internal force distribution and constrained displacement. This result is consistent with the observations of Wan et al. (2024), who, in their research on hybrid timber shells, demonstrated that a system with varying curvature and flexible nodes exhibits superior stability and load-bearing capacity in comparison to classic gridshells [111]. The findings of this study demonstrate that the integration of connections with controlled stiffness within segmental structures facilitates an optimal compromise between mass and stiffness. As demonstrated in the research conducted by Dyvik et al. (2021) [98], analogous relationships were observed. The aforementioned researchers emphasized that gridshells are most effective with uniform load distribution; however, their susceptibility to deformation limits their practical application in large spans [98].

Recent studies have also confirmed that the structural efficiency of timber elements can be significantly increased through the use of composite materials and carbon fiber reinforcements. Experimental analyses have demonstrated that the integration of CFRP layers with glued laminated timber elements results in an enhancement of stiffness and load-bearing capacity, while concurrently maintaining a low structural weight. This finding signifies a pivotal direction for the advancement of hybrid systems in timber architecture [112].

Material optimization of shell structures exerts a direct impact on the reduction in the carbon footprint and consumption of raw materials. Research conducted by Kuda and Petříčková (2021) has demonstrated that modular gridshells have the potential to reduce CO_2_ emissions by 30–40% in comparison with steel structures of equivalent span [113]. The approach employed in this study builds upon this potential by integrating geometric optimization with material analysis. These results are consistent with analyses indicating the importance of geometric and material optimization in reducing energy consumption and CO_2_ emissions in advanced timber structures [114]. The incorporation of environmental parameters, including life cycle analysis (LCA), in forthcoming studies would facilitate a comprehensive evaluation of the sustainability of structures from the design stage to the end of life [115].

Ultimately, the objective is to develop the method in such a manner that it encompasses the modeling of structural behavior in dynamic conditions (i.e., wind, snow, asymmetric loads). Thereafter, the results will be validated on full-scale physical models. The extension of the model with adaptive connections and machine learning algorithms has the potential to facilitate automatic geometry optimization in real time.

It is imperative to acknowledge that the analyses presented herein pertain to ideal models, wherein the influence of assembly errors, variations in timber moisture content, and long-term rheological deformations have been excluded. In real structures, these factors may have a detrimental effect on both stiffness and durability. Notwithstanding the limitations mentioned above, the parametric model is a reliable instrument for conceptual research and provides a point of departure for further experimental and environmental analyses. The findings of earlier studies by the authors further underscore the necessity to corroborate numerical simulations through physical tests of full-scale models, constituting a logical progression of the presented methodology [116].

The results confirm that combining geometric, material, and structural analysis in a single parametric model enables a significant improvement in the efficiency of thin-walled engineered timber shells. Hybrid shell structures exhibit the best stiffness-to-weight ratio and the highest potential for prefabrication and reuse.

From a scientific point of view, this study makes a significant contribution to the development of integrated design methodology for advanced timber structures and sets the direction for further research on automatic shape and material optimization in a parametric design environment. The presented results therefore provide the basis for the development of a universal tool to support the design process of advanced timber structures in the spirit of sustainable development and digital transformation of construction, in line with the principles of contemporary computational timber architecture.

## 5. Conclusions

The research conducted confirms that the application of the proposed design methodology leads to significant material savings and can be an effective tool for optimizing advanced timber structures. Integrated CAD/CAM/CAE design environments now enable the associated control of geometry, structure, and material properties, allowing shape, material, and manufacturing technology data to be combined in a single parametric model. This approach, termed “digital tectonics” in the relevant literature, signifies the contemporary trajectory of development in the fields of architectural and engineering design.

This underscores the necessity for close interdisciplinary collaboration between architects and engineers to engineer systems that marry structural efficiency with spatial and esthetic value. The advent of parametric and algorithmic tools has engendered a paradigm shift in the realm of structural design, enabling the conception of edifices that exhibit a high degree of adaptability to irregular forms while exhibiting minimal material consumption. The utilization of engineered timber and digital manufacturing methodologies has been demonstrated to effectuate a substantial reduction in embodied energy and waste.

It is recommended that further research be conducted on the following points:

(1) The primary objective of this study is to conduct an in-depth analysis of the durability and material optimization of various cladding systems (gridshell, segmented, and hybrid) using parametric tools.
(2) The development and testing of 1:5 and 1:1 scale prototypes is undertaken for the purpose of empirical verification of numerical models.
(3) The third element of the study is an examination of the economic and regulatory aspects that may limit the implementation of such solutions in construction practice.

The long-term objective of the project is the construction of a full-scale prototype pavilion made of engineered timber on the Warsaw University of Life Sciences campus. The purpose of this structure is to demonstrate the practical usefulness of the proposed methodology and its significance for the sustainable development of architecture.

The integration of theoretical rigor with technological innovation establishes a solid foundation for the further development of parametric design in 21st-century architecture, combining technical requirements with artistic expression.

## Figures and Tables

**Table 1:** Experimental engineered wood pavilions (2010–2025).

Project	Design Innovations	Material-Oriented Features	Structural System
ICD/ITKE Research Pavilion (Stuttgart, Germany, 2010)	Bending-active structure using elastic bending; robotically fabricated with computational design; sewn plywood strips for flexibility; unique architectural space from half-torus shape.	Thin birch plywood strips (6.5 mm); anisotropic properties exploited; veneered for industrial sewing; minimal material use for lightweight construction.	Actively bent gridshell [86]
LAGA Exhibition Hall (Schwäbisch Gmünd, Germany, 2014)	Robotically prefabricated segmented timber shell; biomimetic design inspired by natural plate structures; lightweight with free-spanning capability; off-site robotic fabrication of 243 plywood plates.	Beech plywood cassettes; hollow construction for material efficiency; cross-laminated timber for load-bearing elements.	Segmented shell (prefabricated panels) [87]
ICD/ITKE Research Pavilion (Stuttgart, Germany, 2015–2016)	Biomimetic segmented plate shell inspired by beetle elytra; robotic sewing for prefabrication; double-layered structure for efficiency; 151 segments for material-minimizing design.	Laminated beech plywood strips; finger-jointed for strength; anisotropy of wood utilized for structural performance.	Segmented shell (prefabricated panels) [88]
Urbach Tower (Urbach, Germany, 2019)	Self-shaping through controlled drying; no mechanical forming; 14 m slender observation tower; integrative structural design for light weight.	Curved cross-laminated timber (CLT); hygroscopic properties for passive curving; sustainable alternative to energy-intensive methods.	Segmented shell (prefabricated, actively bent panels) [89]
BUGA Wood Pavilion (Heilbronn, Germany, 2019)	Biomimetic segmented shell based on sea urchin plates; robotic fabrication of 376 unique plates; 30 m span with hollow cassettes; designed for reuse.	Hollow beech plywood cassettes; material-efficient with 17,000 finger joints; locally sourced timber.	Segmented gridshell (prefabricated panels) [90]
HygroShell—ITECH Research Pavilion (Chicago, IL, USA, 2023)	Hygroscopic self-shaping panels; on-site curving from flat-packed; 9.5 m long-spanning lightweight shell; adaptive and sustainable building system.	Timber with hygroscopic properties for in situ shaping; lightweight wood structure promoting new material cultures.	Segmented shell (prefabricated, actively bent panels). [91]
ReciproFrame Timber Gridshell (Augsburg, Germany, 2023)	A timber gridshell that forgoes the need for steel nodes or special connectors.	An advanced construction system designed for rapid, cost-effective, and material-efficient assembly and disassembly of reusable structures.	Segmented gridshell (prefabricated panels) [92,93]
Wangen Tower (Wangen in Allgäu, Germany, 2024)	Self-shaped curved CLT for 23 m multi-level tower; torqued 12-sided structure; climbable with inhabitable levels; pioneering timber landmark.	Curved cross-laminated timber (CLT) segments (130 mm thick); hygroscopic self-shaping; renewable, locally sourced timber.	Segmented shell (prefabricated, actively bent panels) [94]
Czech Pavilion for EXPO Osaka (Osaka, Japan, 2025)	Tallest wooden structure in Japan without steel supports; prefabricated panels shipped and assembled; parametric design integrating local materials; continuous spiral ramp.	Cross-laminated timber (CLT) panels; PEFC-certified wood; glass facade with insulation panels; sustainable and resource-effective.	Slab and column–slab system [95]

**Table 2:** Comparison of structural systems.

Structural System	Key Characteristics	Material Efficiency (kg/m^2^)	Stiffness (Deflection Under 1.9 kPa Load)	Literature Examples
DISCRETE GRIDSHELL	Elastic deformation of timber laths into double-curved lattices; orthogonal or diagonal meshes.	40–60	Moderate (e.g., 2–5 mm/m span); relies on curvature for stability.	Mannheim Pavilion (1975): 50 mm × 50 mm hemlock laths, 60 m span.
SEGMENTED SHELL	Modular planar panels (e.g., CLT cassettes) assembled into free-form envelopes; reversible joints.	25–35	High (e.g., <2 mm/m); enhanced by topology optimization.	BUGA Wood Pavilion: hollow LVL cassettes, 41% lighter than CLT.
RIBBED SHELL (HYBRID)	Integration of gridshell lattices with segmented panels or bracing; reciprocal nodes.	20–30	Superior (e.g., 2.3–2.4 mm under 300 kg); 60% material reduction.	Reciproframe Gridshell: plywood frames with spruce bracing, robotic fabrication.

**Table 3:** Comparison of structural properties of 10 mm and 20 mm plywood.

**Parameter**	**~10 mm (9–12 mm)**	**~20 mm (18–21 mm)**
Density (kg/m^3^)	670–750	670–750
Modulus of Elasticity (MOE) in bending (N/mm^2^)	10,000–12,000 (e.g., 12,805 at 9 mm, 11,975 at 12 mm)	8700–11,000 (e.g., 11,069 at 18 mm, 10,795 at 21 mm)
Bending Strength (N/mm^2^)	53–57 (e.g., 56.9 at 9 mm, 53.2 at 12 mm)	48–49 (e.g., 49.2 at 18 mm, 48.0 at 21 mm)
Shear Strength in panel shear (N/mm^2^)	10.0	10.0
Shear Capacity (kN/m or N/mm width)	15–21 (e.g., 133 N/mm at 9 mm, 325 at 12 mm; 98–126 kN/m)	27–33 (e.g., 663 N/mm at 19 mm, 838 at 21 mm; 182–210 kN/m approximated)
Tension Strength (N/mm^2^)	43–45 (e.g., 44.6 at 9 mm, 43.3 at 12 mm)	41–42 (e.g., 42.0 at 18 mm, 41.6 at 21 mm)
Compression Strength (N/mm^2^)	Varies by grade, e.g., 20–45 MPa (F8–F17)	Varies by grade, e.g., 20–45 MPa (F8–F17)
Bending Capacity (N/mm width, F8 grade example)	250–363 (at 9–12 mm)	950–1288 (at 19–21 mm)

## Data Availability

The original contributions presented in this study are included in the article. Further inquiries can be directed to the corresponding author.

## References

[1] Giannotas G., Kamperidou V., Stefanidou M., Kampragkou P., Liapis A., Barboutis I. (2022). Utilization of Tree-Bark in Cement Pastes. J. Build. Eng..

[2] Ruuska A., Häkkinen T. (2014). Material Efficiency of Building Construction. Buildings.

[3] González-Retamal M., Forcael E., Saelzer-Fuica G., Vargas-Mosqueda M. (2022). From Trees to Skyscrapers: Holistic Review of the Advances and Limitations of Multi-Storey Timber Buildings. Buildings.

[4] Starzyk A., Marchwiński J., Milošević V. (2025). Circular Wood Construction in a Sustainable Built Environment: A Thematic Review of Gaps and Emerging Topics. Sustainability.

[5] Ren H., Bahrami A., Cehlin M., Wallhagen M. (2024). Flexural Behavior of Cross-Laminated Timber Panels with Environmentally Friendly Timber Edge Connections. Buildings.

[6] Menges A., Schwinn T., Krieg O.D. (2016). Advancing Wood Architecture.

[7] Weinand Y. (2016). Advanced Timber Structures: Architectural Designs and Digital Dimensioning.

[8] Søndergaard A., Amir O., Eversmann P., Piskorec L., Stan F., Gramazio F., Kohler M. (2016). Topology Optimization and Robotic Fabrication of Advanced Timber Space-Frame Structures. Robotic Fabrication in Architecture, Art and Design 2016.

[9] Bertetto A.M., Gabriele S., Marmo F., Micheletti A. (2020). Shell and Spatial Structures: Between New Developments and Historical Aspects. Curved Layer. Struct..

[10] Bechert S., Sonntag D., Aldinger L., Knippers J. (2021). Integrative Structural Design and Engineering Methods for Segmented Timber Shells—BUGA Wood Pavilion. Structures.

[11] Formichetti G., Matteoni M., Pampanin S. Development of a Holistic Parametric Framework for Multi-Performance Life-Cycle Evaluation of Post-Tensioned Timber Buildings. Proceedings of the 9th ECCOMAS Thematic Conference on Computational Methods in Structural Dynamics and Earthquake Engineering.

[12] Trautz M., Grizmann D., Seiter A., Moreno Gata K., Pranjic A., Saez D. (2023). Design and Natural Materials—Innovative Approaches for a Sustainable Future Architecture and Structural Engineering. Proceedings of the Creative Construction Conference.

[13] Porras E., Esenarro D., Chang L., Morales W., Vargas C., Sucasaca J. (2024). Toward Cost-Effective Timber Shell Structures through the Integration of Computational Design, Digital Fabrication, and Mechanical Integral ‘Half-Lap’ Joints. Buildings.

[14] Valdés M.D., Bohórquez M.G. (2023). Novel Proposal of Bio-Based Sewing Timber Joint: Learning from Diatoms. J. Build. Mater. Sci..

[15] Victoria L.C., Aquino C.D., Branco J.M., Miguel L.F.F. (2025). Numerical Analysis of Cross-Laminated Timber (CLT) Buildings: A Parametric Study on Steel Connectors When Subjected to Seismic Loading Under Eurocode 8 and NBR 15421. Buildings.

[16] Giuriani E., Marini A. (2008). Wooden Roof Box Structure for the Anti-Seismic Strengthening of Historic Buildings. Int. J. Archit. Herit..

[17] Sharifi J., Sharifi Z., Berg S., Ekevad M. (2021). Diaphragm Shear and Diagonal Compression Testing of Cross-Laminated Timber. SN Appl. Sci..

[18] Heesterman M. (2019). The Robotic Craftsman: Robotic Fabrication for Complex Timber Connections. Master’s Thesis.

[19] Starzyk A., Cortiços N.D., Duarte C.C., Łacek P. (2025). Timber Architecture for Sustainable Futures: A Critical Review of Design and Research Challenges in the Era of Environmental and Social Transition. Buildings.

[20] Starzyk A., Łacek P., Koda E., Daria Vaverková M., Reddy K.R., Agnihotri A.K. (2025). Wood as an Architectural and Construction Material in the Circular Economy. Solid Waste and Recycling.

[21] Golański M. (2022). Advances in Digital Design and Fabrication of Wooden Architecture. Archit. Artibus.

[22] Golański M. (2023). Analogue and Computational Form-Finding Techniques in Shell Structures Design. Archit. Artibus.

[23] Juchimiuk J. (2022). Energy Metamorphosis of Cities and Buildings. Archit. Artibus.

[24] Juchimiuk J. (2022). Renewable Energy Sources in Architecture of the World Expo. Archit. Artibus.

[25] Juchimiuk J., Golański M. (2025). Factors and Drivers of Architectural Form Modelling in Aspect of Climate Change. Inżynieria Bezpieczeństwa Obiektów Antropog..

[26] Kensek K. (2015). Visual Programming for Building Information Modeling: Energy and Shading Analysis Case Studies. J. Green Build..

[27] Silva L.S., Najjar M.K., Tam V.W., Haddad A.N., Amario M. (2025). An Integrated Technical Process of Building Information Modeling Enabled by Visual Programming Language: An Optimized Computational Building Solution for Energy Efficiency. Sustain. Mater. Technol..

[28] Ghannad P., Lee Y.-C., Dimyadi J., Solihin W. (2019). Automated BIM Data Validation Integrating Open-Standard Schema with Visual Programming Language. Adv. Eng. Inform..

[29] Nawari N. (2012). BIM Standardization and Wood Structures. Comput. Civ. Eng..

[30] Abushwereb M., Liu H., Al-Hussein M. (2019). A Knowledge-Based Approach towards Automated Manufacturing-Centric BIM: Wood Frame Design and Modelling for Light-Frame Buildings. Modul. Offsite Constr. MOC Summit Proc..

[31] Lobos Calquin D., Mata R., Correa C., Nuñez E., Bustamante G., Caicedo N., Blanco Fernandez D., Díaz M., Pulgar-Rubilar P., Roa L. (2024). Implementation of Building Information Modeling Technologies in Wood Construction: A Review of the State of the Art from a Multidisciplinary Approach. Buildings.

[32] Charleson A. (2014). Structure as Architecture: A Source Book for Architects and Structural Engineers.

[33] Macdonald A.J. (2018). Structure and Architecture.

[34] Piętocha A. (2024). The BREEAM, the LEED and the DGNB Certifications as an Aspect of Sustainable Development. Acta Sci. Pol. Archit..

[35] Piętocha A., Li W., Koda E. (2025). The Vertical City Paradigm as Sustainable Response to Urban Densification and Energy Challenges: Case Studies from Asian Megacities. Energies.

[36] Fleischmann M., Menges A. (2011). Icd/Itke Research Pavilion: A Case Study of Multi-Disciplinary Collaborative Computational Design. Proceedings of the Computational Design Modelling: Proceedings of the Design Modelling Symposium Berlin.

[37] Menges A., Knippers J. (2020). Architecture Research Building: ICD/ITKE 2010–2020.

[38] Menges A. (2017). Design Computation and Material Culture. Lineament: Material, Representation and the Physical Figure in Architectural Production.

[39] Knippers J., Kropp C., Menges A., Sawodny O., Weiskopf D. (2021). Integrative Computational Design and Construction: Rethinking Architecture Digitally. Civ. Eng. Des..

[40] Weinand Y. (2009). Innovative Timber Constructions. J. Int. Assoc. Shell Spat. Struct..

[41] Gamerro J., Bocquet J.-F., Weinand Y. Future Challenges and Need for Research in Timber Engineering. Proceedings of the 8th Workshop of COST Action FP1402 “Basis of Structural Timber Design-from research to standards”.

[42] Weinand Y. (2016). Timber Fabric Structures: Innovative Wood Construction. Advancing Wood Architecture.

[43] Willmann J., Knauss M., Bonwetsch T., Apolinarska A.A., Gramazio F., Kohler M. (2016). Robotic Timber Construction—Expanding Additive Fabrication to New Dimensions. Autom. Constr..

[44] Apolinarska A.A., Knauss M., Gramazio F., Kohler M. (2016). The Sequential Roof. Advancing Wood Architecture.

[45] Krieg O.D., Lang O. (2019). Adaptive Automation Strategies for Robotic Prefabrication of Parametrized Mass Timber Building Components. Proceedings of the International Symposium on Automation and Robotics in Construction.

[46] Apolinarska A., Bärtschi R., Furrer R., Gramazio F., Kohler M. (2016). Mastering the Sequential Roof. Adv. Archit. Geom..

[47] Tamke M. (2013). CITA: Working for and with Material Performance. Serbian Archit. J..

[48] Thomsen M., Tamke M., Ayres P., Nicholas P. (2015). CITA Works.

[49] Thomsen M.R. (2021). CITA Complex Modelling.

[50] Carpo M. (2016). Parametric Notations: The Birth of the Non-Standard. Archit. Des..

[51] Fatah gen Schieck A., Hanna S. (2007). Embedded, Embodied, Adaptive: Architecture and Computation.

[52] Sheine J., Fretz M., O’Halloran S., Gershfeld M., Stenson J. (2023). Mass Timber Panelized Workforce Housing in Oregon, U.S..

[53] Heikkinen P., Tidwell P. (2017). Liina Shelter, Wood Program. The Design-Build Studio.

[54] Tidwell P., Heikkinen P. (2019). Timber Joints at the Aalto University Wood Program: Designing Through Experimentation. Rethinking Wood: Future Dimensions of Timber Assembly.

[55] Heikkinen P. Wood Program 30 Years. Proceedings of the Aalto ARTS Student Show.

[56] Pedersen J. (2023). Parawood: Framework for on-Site Robotic Timber Fabrication. Ph.D. Thesis.

[57] Gatóo A., Ramage M.H., Bakker R. (2024). Ephemeral Walls for Natural, Flexible Living. Proceedings of the IOP Conference Series: Earth and Environmental Science.

[58] Reynolds T., Feldmann A., Ramage M., Chang W.-S., Harris R., Dietsch P. Design Parameters for Lateral Vibration of Multi-Storey Timber Buildings. Proceedings of the International Network on Timber Engineering Research Meeting 49.

[59] Robeller C., Von Haaren N. (2020). Recycleshell: Wood-Only Shell Structures Made from Cross-Laminated Timber (CLT) Production Waste. J. Int. Assoc. Shell Spat. Struct..

[60] Krieg O.D., Schwinn T., Menges A., Li J.-M., Knippers J., Schmitt A., Schwieger V., Block P., Knippers J., Mitra N.J., Wang W. (2015). Biomimetic Lightweight Timber Plate Shells: Computational Integration of Robotic Fabrication, Architectural Geometry and Structural Design. Advances in Architectural Geometry 2014.

[61] Redkin A., Baranovskii M., Tarasov V. (2016). Steel and Composite Structural Shells in the Construction of High-Rise Building. MATEC Web Conf..

[62] Scholzen A., Chudoba R., Hegger J. (2015). Thin-walled Shell Structures Made of Textile-reinforced Concrete: Part I: Structural Design and Construction. Struct. Concr..

[63] Kushwaha V., Mishra R., Kumar S. (2015). A Comprehensive Study for Economic and Sustainable Design of ThinShell Structure for Different Loading Conditions. https://d1wqtxts1xzle7.cloudfront.net/54685338/IRJET-V3I118-libre.pdf?1507709905=&response-content-disposition=inline%3B+filename%3DA_Comprehensive_Study_for_Economic_and_S.pdf&Expires=1767595997&Signature=VD1DRh-YVvx38M1~KxaQb58F6kXTBDlYiaVp-dyw6z1C3g~i0crJHdnQBJm4ztnKQNPpEaUXmFMAWIpkEy~iF0xwydHMhhyH4S12wMv7iybue3J1n3kElHH-jc9PatSBw0ELsraEHtcmNvS9NXGwdZRawjAZouYnNjVca5XhAW8kYpXrOlEn0VQci3UC1607jG310IEKqi8uTe87BSOn-BR0m4eGCnx08i2c7~mfdSzE54vDQgENi6mJx7t52H31hcnvBg~RMTvjIlqMOk1-lyuOVm84XF5jUGVTT5KhdSgG40Ie-MDcr2ja3Nxj2dJVF72Q7AZIxOklLw4HcdwFvA__&Key-Pair-Id=APKAJLOHF5GGSLRBV4ZA.

[64] Hawkins W., Orr J., Shepherd P., Tim I., Bregulla J. Thin-Shell Textile-Reinforced Concrete Floors for Sustainable Buildings. Proceedings of the IASS Annual Symposia.

[65] Veltkamp M. (2007). Free Form Structural Design: Schemes, Systems & Prototypes of Structures for Irregular Shaped Buildings.

[66] Veltkamp M. (2010). Structural Optimization of Free Form Framed Structures in Early Stages of Design. Proceedings of the Symposium of the International Association for Shell and Spatial Structures.

[67] Misztal B. (2018). Wooden Domes.

[68] Misztal B. (2017). Gridshell, Ribbed–Shell Domes. Wooden Domes: History and Modern Times.

[69] Misztal B. (2017). Multi-Shell Domes. Wooden Domes: History and Modern Times.

[70] Misztal B. (2017). Ribbed Domes. Wooden Domes: History and Modern Times.

[71] Misztal B. (2017). Selected Elements of the Dome Building History. Wooden Domes: History and Modern Times.

[72] Misztal B. (2017). Selected Examples of Domes from Glued Laminated Timber. Wooden Domes: History and Modern Times.

[73] Misztal B. (2025). On the Variability in Time of the Longitudinal Modulus of Elasticity E and the Traverse Modulus of Elasticity G and Their Impact on the Rigidity of Timber Structures. Architectus.

[74] Misztal B. (2017). Shell Domes. Wooden Domes: History and Modern Times.

[75] Apóstol Á. Club Tahira Caracas 2025. https://clubtachiracaracas.com/.

[76] Golański M., Januszkiewicz K. (2017). Free-Form Wooden Architectural Envelopes in Terms of Energy Efficiency. Int. Multidiscip. Sci. GeoConf. SGEM.

[77] Januszkiewicz K., Banachowicz M. (2017). Nonlinear Shaping Architecture Designed with Using Evolutionary Structural Optimization Tools. Proceedings of the IOP Conference Series: Materials Science and Engineering.

[78] Mouchart M., Orsi R. (2016). Building a Structural Model: Parameterization and Structurality. Econometrics.

[79] Tarczewski R. (2013). Natural and Geometrical Prototypes of Organic Forms in Architecture. Proceedings of the IASS Annual Symposia.

[80] Chilton J., Tang G. (2016). Timber Gridshells: Architecture, Structure and Craft.

[81] D’Amico B., Kermani A., Zhang H., Pugnale A., Colabella S., Pone S. (2015). Timber Gridshells: Numerical Simulation, Design and Construction of a Full Scale Structure. Structures.

[82] Schling E., Wan Z., Wang H., D’Acunto P. (2023). Asymptotic Geodesic Hybrid Timber Gridshell. Advances in Architectural Geometry 2023.

[83] Green M., Taggart J. (2025). Tall Wood Buildings: Design.

[84] Dickson M., Parker D. (2014). Sustainable Timber Design.

[85] Armpriest D., Manrique C. (2019). Structures and Architecture Integration in a Best Use of Wood Design Competition Studio. Structures and Architecture-Bridging the Gap and Crossing Borders.

[86] Krieg O.D. ICD/ITKE Research Pavilion 2010. https://eumiesawards.com/heritageobject/icditke-research-pavilion/.

[87] Menges A., Knippers J. (2014). Landesgartenschau Exhibition Hall 2014. https://www.itke.uni-stuttgart.de/research/built-projects/landesgartenschau-exhibition-hall-2014/.

[88] Menges A., Knippers J. (2016). ICD/ITKE Research Pavilion 2015-16. https://www.itke.uni-stuttgart.de/research/icd-itke-research-pavilions/icd-itke-research-pavilion-2015-16/.

[89] Menges A., Knippers J. (2019). Urbach Tower. https://www.icd.uni-stuttgart.de/projects/remstal-gartenschau-2019-urbach-turm/.

[90] Menges A., Knippers J. BUGA Wood Pavilion 2019. https://www.icd.uni-stuttgart.de/projects/buga-wood-pavilion-2019/.

[91] Menges A., Knippers J. (2023). HygroShell-ITECH Research Pavilion, Chicago Architecture Biennial 2023. https://www.icd.uni-stuttgart.de/projects/hygroshell/.

[92] Adelzadeh A., Karimian H., Robeller C. ReciproFrame Timber Gridshell: From CAM Data Interface Modeling to Operating Industrial Joinery Machine for Scaling up Reusable Timber Structures. Proceedings of the CAADRIA 2024—Accelerated Design.

[93] Adelzadeh A., Karimian-Aliabadi H., Åhlund K., Robeller C. ReciprocalShell: A Hybrid Timber System for Robotically-Fabricated Lightweight Shell Structures. Proceedings of the 41st Conference on Education and Research in Computer Aided Architectural Design in Europe.

[94] Menges A., Knippers J. (2024). Wangen Tower. https://www.icd.uni-stuttgart.de/projects/wangen-tower/.

[95] Gabaš M., Beránek T., Slováková N., Šváchová T., Nikerle R., Jireš K., Studio L., ZAN Studio World Expo 2025 in Osaka—Apropos Architects 2025. https://aproposarchitects.com/project/czech-pavilion-for-the-world-expo-2025-in-osaka/.

[96] Lehmann S. (2024). Multihalle Pavilion: The Renovation of an Innovative, Experimental Structure From the 1970s. Mass Timber Constr. J..

[97] Mertens M. (2020). Der Gestrandete Wal: Das Baudenkmal Multihalle. Denkmalpfl. Baden-Württ. Landesdenkmalpflege.

[98] Dyvik S.H., Manum B., Rønnquist A. (2021). Gridshells in Recent Research—A Systematic Mapping Study. Appl. Sci..

[99] Soti R., Ho T.X., Sinha A. (2021). Structural Performance Characterization of Mass Plywood Panels. J. Mater. Civ. Eng..

[100] Wang T., Wang Y., Crocetti R., Wålinder M. (2022). In-Plane Mechanical Properties of Birch Plywood. Constr. Build. Mater..

[101] Engineering ToolBox Wood, Panel and Structural Timber Products—Mechanical Properties 2011. https://www.engineeringtoolbox.com/timber-mechanical-properties-d_1789.html.

[102] Cai Z., Ross R.J. (2010). Mechanical Properties of Wood-Based Composite Materials.

[103] Finnish Forest Industries Federation (2002). Handbook of Finnish Plywood.

[104] Koskisen Group (2023). Handbook of Koskisen Plywood.

[105] Dasari S.K., Fantuzzi N., Trovalusci P., Panei R. (2022). Computational Approach for Form-Finding Optimal Design. Archit. Struct. Constr..

[106] Teschemacher T., Bauer A.M., Aristio R., Meßmer M., Wüchner R., Bletzinger K.-U. (2022). Concepts of Data Collection for the CAD-Integrated Isogeometric Analysis. Eng. Comput..

[107] Berdos Y., Agkathidis A., Brown A. (2020). Architectural Hybrid Material Composites: Computationally Enabled Techniques to Control Form Generation. Archit. Sci. Rev..

[108] Hua H., Hovestadt L., Tang P. (2020). Optimization and Prefabrication of Timber Voronoi Shells. Struct. Multidiscip. Optim..

[109] Schling E., Wan Z., D’Acunto P. (2023). Design and Construction of an Asymptotic Geodesic Timber Gridshell. Proceedings of the XI Textile Composites and Inflatable Structures.

[110] Fairclough H.E., Bolbotowski K., He L., Liew A., Gilbert M. (2025). Topology and Geometry Optimization of Grid-Shells under Self-Weight Loading. arXiv.

[111] Wan Z., D’Acunto P., Schling E. (2024). Structural Behaviour of Asymptotic Geodesic Hybrid Timber Gridshells. Eng. Struct..

[112] Bakalarz M.M. (2024). Mechanical Properties of Full-Scale Wooden Beams Strengthened with Carbon-Fibre-Reinforced Polymer Sheets. Materials.

[113] Kuda D., Petříčková M. (2021). Modular Timber Gridshells. J. Sustain. Archit. Civ. Eng..

[114] Starzyk A., Rybak-Niedziółka K., Nowysz A., Marchwiński J., Kozarzewska A., Koszewska J., Piętocha A., Vietrova P., Łacek P., Donderewicz M. (2024). New Zero-Carbon Wooden Building Concepts: A Review of Selected Criteria. Energies.

[115] Golański M. (2012). Wybór Materiałów Budowlanych w Kontekście Efektywności Energetycznej i Wpływu Środowiskowego. Bud. Inżynieria Sr..

[116] Starzyk A., Cortiços N.D., Duarte C.C., Łacek P. (2024). Environmental and Architectural Aspects of Wooden Construction: A Comparative Analysis of Selected Issues of Single-Family Housing in Poland and Portugal. Acta Sci. Pol. Archit..

